# From immune response to mental health: neutrophils in schizophrenia

**DOI:** 10.1097/MS9.0000000000002744

**Published:** 2024-11-12

**Authors:** Emmanuel Ifeanyi Obeagu, Getrude Uzoma Obeagu

**Affiliations:** a Department of Biomedical and Laboratory Science, Africa University, Zimbabwe; b School of Nursing Science, Kampala International University, Ishaka, Uganda

**Keywords:** immune response, inflammation, neutrophils, psychiatric disorders, schizophrenia

## Abstract

Schizophrenia is a multifaceted psychiatric disorder with a complex etiology that includes genetic, environmental, and immunological factors. Emerging research has increasingly focused on the role of the immune system, particularly neutrophils, in the pathogenesis of schizophrenia. Neutrophils, the most abundant type of white blood cells, are crucial in the body’s defense mechanisms against infections and in the regulation of inflammation. This review explores the evolving evidence that implicates neutrophils in schizophrenia, highlighting their potential contribution to the disorder’s onset and progression through mechanisms such as neuroinflammation and oxidative stress. Recent studies have demonstrated elevated neutrophil counts and neutrophil-to-lymphocyte ratios (NLR) in individuals with schizophrenia, correlating with the severity of psychotic symptoms and cognitive impairments. Furthermore, genetic and molecular analyses have revealed abnormalities in neutrophil function and immune-related gene expression in schizophrenia patients. These findings suggest that neutrophil dysfunction and the resulting chronic inflammation could play a significant role in the disorder’s pathophysiology, affecting neuronal function, and contributing to the clinical manifestations of schizophrenia. Neutrophil-related biomarkers, such as NLR, could aid in the diagnosis and monitoring of disease progression. Additionally, targeting neutrophil activity and associated inflammatory pathways presents a promising avenue for developing new therapeutic interventions. This review underscores the need for further research to elucidate the precise mechanisms by which neutrophils influence schizophrenia and to explore potential treatments that could improve outcomes for patients.

## Introduction

Schizophrenia is a severe and chronic psychiatric disorder that affects ~ 1% of the global population. It is characterized by a wide range of symptoms, including hallucinations, delusions, disorganized thinking, and significant cognitive deficits. The etiology of schizophrenia is multifactorial, involving a complex interplay of genetic, environmental, and neurobiological factors. Despite extensive research, the exact mechanisms underlying the disorder remain elusive, and there is a pressing need to explore new pathways that could offer insights into its pathophysiology and treatment^[1-4]^. In recent years, the immune system has emerged as a significant area of interest in the study of schizophrenia. Historically, psychiatric disorders were considered to be purely neurochemical or neurodevelopmental in nature. However, accumulating evidence suggests that immune dysregulation and inflammation may play a crucial role in the onset and progression of schizophrenia. Among the various components of the immune system, neutrophils have gained attention due to their potential involvement in neuroinflammatory processes associated with psychiatric disorders^[5,6]^. Neutrophils are the most abundant type of white blood cells in the human body, representing a key component of the innate immune system. They are the first responders to infection and injury, playing a vital role in the body’s defense mechanisms through processes such as phagocytosis, degranulation, and the formation of neutrophil extracellular traps (NETs). In addition to their antimicrobial activities, neutrophils are also involved in the regulation of inflammation and tissue repair, making them crucial players in both acute and chronic inflammatory responses^[7,8]^.

**Highlights**

- Emerging role: Neutrophils, typically in infection defense, are now implicated in schizophrenia’s immune dysregulation.
- Neuroinflammation nexus: Neutrophils contribute via cytokines and oxidative stress, impacting neuronal integrity and cognitive functions.
- Genetic insights: Variants in immune genes affect neutrophil activity, influencing schizophrenia susceptibility and severity.
- Environmental influence: Prenatal infections and stressors exacerbate immune dysregulation, altering neutrophil function in schizophrenia.
- Therapeutic potential: Targeting neutrophil-mediated inflammation and oxidative stress offers new avenues for schizophrenia treatment and personalized medicine approaches.

The concept of neuroinflammation as a contributor to psychiatric disorders, including schizophrenia, has gained substantial support over the past decade. Neuroinflammation refers to the activation of the brain’s innate immune system in response to various insults, leading to the release of proinflammatory cytokines, chemokines, and other inflammatory mediators. This inflammatory milieu can disrupt normal brain function, contributing to the development of psychiatric symptoms. Neutrophils, as key effector cells in inflammation, may thus have a significant impact on neuroinflammatory processes in schizophrenia^[9]^. Recent studies have reported elevated levels of neutrophils and increased neutrophil-to-lymphocyte ratios (NLR) in patients with schizophrenia^[10,11]^. These findings suggest that an ongoing inflammatory process involving neutrophils may be present in schizophrenia, correlating with the severity of symptoms and cognitive impairments. The exact role of neutrophils in the pathophysiology of schizophrenia is not fully understood, but several potential mechanisms have been proposed. One potential mechanism is the disruption of the blood—brain barrier (BBB) by inflammatory mediators released by neutrophils. The BBB is a critical structure that protects the central nervous system (CNS) from peripheral immune cells and pathogens. However, in conditions of inflammation, neutrophils can traverse the BBB and enter the CNS, where they release proinflammatory cytokines and reactive oxygen species (ROS). This can lead to neuronal damage and exacerbate the clinical manifestations of schizophrenia^[12]^.

Another mechanism involves oxidative stress, which is a state of imbalance between the production of ROS and the body’s ability to detoxify these reactive intermediates. Neutrophils produce ROS as part of their antimicrobial activities, but excessive ROS production can result in oxidative damage to neural tissues. Oxidative stress has been implicated in the pathophysiology of schizophrenia, potentially contributing to neurodevelopmental abnormalities and cognitive deficits^[12]^. Genetic studies have also highlighted the involvement of immune-related genes in schizophrenia. Polymorphisms in genes related to immune function and inflammation have been identified in individuals with schizophrenia, suggesting a genetic predisposition to immune dysregulation. Moreover, molecular analyses have shown altered expression of genes involved in neutrophil activation and function in schizophrenia patients, further supporting the link between neutrophils and the disorder^[13,14]^.

## Aim

The aim of this review is to critically examine and synthesize the current evidence on the role of neutrophils in the pathophysiology of schizophrenia.

## Rationale

The exploration of immune system involvement in psychiatric disorders, particularly schizophrenia, has gained substantial momentum in recent years. While traditionally considered to be a neurochemical and neurodevelopmental disorder, emerging evidence suggests that immune dysregulation and inflammation play critical roles in the pathophysiology of schizophrenia. Among the various immune cells, neutrophils, the most abundant type of white blood cells, have been increasingly implicated in neuroinflammatory processes that may contribute to the disorder. Neutrophils are key players in the innate immune response, responsible for defending against infections and mediating inflammatory responses. Their involvement in chronic inflammation has been well-documented in various physical health conditions, but their role in mental health disorders, particularly schizophrenia, is less understood. Recent studies have reported elevated neutrophil counts and increased neutrophil-to-lymphocyte ratios (NLR) in individuals with schizophrenia, suggesting a state of heightened immune activation and inflammation in these patients. Second, elucidating the mechanisms through which neutrophils contribute to schizophrenia could identify novel biomarkers for early diagnosis and disease monitoring, which are currently lacking. Third, targeting neutrophil-related pathways could open new avenues for therapeutic interventions, addressing the underlying inflammatory processes that may exacerbate schizophrenia symptoms. By synthesizing current knowledge on neutrophils in schizophrenia, this review aims to bridge gaps in our understanding and highlight the significance of the immune system in mental health. Given the complexity of schizophrenia and the multifactorial nature of its pathogenesis, a comprehensive examination of the role of neutrophils could pave the way for more holistic approaches to treatment and management, ultimately improving outcomes for individuals affected by this debilitating disorder.

## The immune-mind connection of neutrophils in schizophrenia

Traditionally, the focus has been on genetic and neurochemical abnormalities. However, recent research has increasingly implicated the immune system in the pathogenesis of schizophrenia, highlighting the importance of immune cells, particularly neutrophils, in understanding the disorder’s complexity^[15]^. Neutrophils are a fundamental component of the innate immune system, serving as the first line of defense against infections^[16]^. They are involved in various immune responses, including phagocytosis, degranulation, and the formation of neutrophil extracellular traps (NETs). Beyond their antimicrobial functions, neutrophils play a crucial role in regulating inflammation and tissue repair, processes that are vital for maintaining homeostasis but can also contribute to disease when dysregulated. Neuroinflammation is increasingly recognized as a significant factor in the pathophysiology of schizophrenia^[17]^. The central nervous system (CNS) can become inflamed due to various factors, including infections, stress, and autoimmune responses. Neutrophils, as key mediators of inflammation, can infiltrate the CNS, particularly when the blood–brain barrier (BBB) is compromised. This infiltration can lead to the release of proinflammatory cytokines and reactive oxygen species (ROS), exacerbating neuronal damage and potentially contributing to the symptoms of schizophrenia.

Several studies have reported elevated neutrophil counts and increased neutrophil-to-lymphocyte ratios (NLR) in patients with schizophrenia^[18,19]^. These findings indicate an ongoing inflammatory process and suggest that neutrophils may play a role in the disorder’s severity and progression. Higher NLR values have been associated with more severe psychotic symptoms and cognitive deficits, underscoring the potential impact of neutrophilmediated inflammation in schizophrenia. Research suggests that neutrophils in individuals with schizophrenia may exhibit functional abnormalities^[20]^. These can include impaired chemotaxis, reduced phagocytic activity, and altered degranulation. Such dysfunctions can lead to an inadequate immune response, persistent inflammation, and an imbalance in immune regulation, all of which could contribute to the pathophysiology of schizophrenia. Genetic studies have identified polymorphisms in immune-related genes among individuals with schizophrenia, pointing to a genetic predisposition to immune dysregulation^[13,21]^. Molecular analyses have further revealed altered expression of genes involved in neutrophil activation and function. These genetic and molecular insights provide strong evidence for the involvement of neutrophils in the immune response abnormalities observed in schizophrenia.

Several mechanisms have been proposed to explain how neutrophils might influence the development and progression of schizophrenia. One key mechanism is the disruption of the blood–brain barrier by inflammatory mediators released by neutrophils. This disruption allows more immune cells to enter the CNS, increasing neuroinflammation and neuronal damage. Additionally, oxidative stress caused by excessive ROS production from neutrophils can damage neural tissues and affect neurodevelopment, contributing to schizophrenia’s cognitive and behavioral symptoms. The association between neutrophils and schizophrenia has significant clinical implications. Neutrophilrelated biomarkers, such as NLR, could serve as valuable tools for diagnosing schizophrenia and monitoring its progression. Moreover, therapeutic strategies targeting neutrophil function and the inflammatory pathways they modulate may offer new avenues for treatment. Anti-inflammatory agents, antioxidants, and therapies aimed at restoring BBB integrity are potential areas for therapeutic development^[21]^.

## Inflammation and oxidative stress of neutrophils in schizophrenia

While traditionally attributed to genetic and neurochemical factors, recent research highlights the significant role of immune system dysregulation, particularly inflammation and oxidative stress mediated by neutrophils, in the pathophysiology of schizophrenia. Neutrophils are the most abundant type of white blood cells and are crucial players in the innate immune system^[22]^. They act as the body’s first line of defense against infections by engaging in phagocytosis, degranulation, and the formation of neutrophil extracellular traps (NETs). In addition to their antimicrobial functions, neutrophils are key regulators of inflammation. Upon activation, they release a variety of proinflammatory cytokines and chemokines, which recruit and activate other immune cells, amplifying the inflammatory response. Neuroinflammation, characterized by the activation of the brain’s innate immune system, is increasingly recognized as a critical component in the development and progression of schizophrenia^[23]^. Elevated levels of proinflammatory cytokines, such as interleukin-6 (IL-6) and tumor necrosis factor-alpha (TNF-α), have been observed in the blood and cerebrospinal fluid of patients with schizophrenia^[24]^. These cytokines can cross the blood–brain barrier (BBB) and induce inflammatory responses within the central nervous system (CNS), potentially leading to neuronal damage and the exacerbation of psychiatric symptoms.

Oxidative stress, defined as an imbalance between the production of reactive oxygen species (ROS) and the body’s ability to detoxify these reactive intermediates, plays a significant role in the pathophysiology of schizophrenia^[25]^. Neutrophils are a major source of ROS, which they produce as part of their antimicrobial activity. However, excessive ROS production can lead to oxidative damage to cellular structures, including lipids, proteins, and DNA. This oxidative damage can disrupt neuronal function and integrity, contributing to the cognitive deficits and neurodevelopmental abnormalities observed in schizophrenia. Several studies have reported elevated neutrophil counts and increased neutrophil-to-lymphocyte ratios (NLR) in patients with schizophrenia, indicating a state of heightened immune activation and inflammation^[26,27]^. Higher NLR values have been associated with more severe psychotic symptoms and cognitive impairments. Additionally, research suggests that neutrophils in individuals with schizophrenia may exhibit functional abnormalities, such as impaired chemotaxis and reduced phagocytic activity, leading to persistent inflammation and oxidative stress. The BBB is a critical structure that protects the CNS from peripheral immune cells and pathogens. In conditions of inflammation, neutrophils can release enzymes and ROS that disrupt the integrity of the BBB, allowing immune cells and proinflammatory mediators to enter the CNS. This increased permeability can exacerbate neuroinflammation and neuronal damage, influencing the clinical presentation of schizophrenia. Studies have shown that patients with schizophrenia have increased BBB permeability, which may be partly mediated by neutrophil activity.

Genetic studies have identified polymorphisms in immunerelated genes among individuals with schizophrenia, suggesting a genetic predisposition to immune dysregulation. For instance, polymorphisms in genes involved in the regulation of neutrophil function and inflammatory responses have been associated with an increased risk of schizophrenia. Molecular analyses have also revealed altered expression of genes related to neutrophil activation and ROS production in schizophrenia patients, further supporting the link between neutrophils, inflammation, and oxidative stress in the disorder^[21,28]^. Neutrophil-related biomarkers, such as NLR, could serve as valuable tools for diagnosing schizophrenia and monitoring its progression. Additionally, targeting neutrophil function and the inflammatory pathways they modulate may offer new therapeutic approaches. Anti-inflammatory agents, antioxidants, and therapies aimed at restoring BBB integrity are potential areas for therapeutic development. Given the involvement of neutrophils in inflammation and oxidative stress in schizophrenia, several therapeutic strategies can be considered. Anti-inflammatory drugs that specifically target neutrophil activity or inhibit the release of proinflammatory cytokines could help mitigate neuroinflammation. Antioxidant therapies aimed at reducing ROS levels and protecting against oxidative damage may also prove beneficial. Moreover, treatments designed to restore BBB integrity could prevent the infiltration of peripheral immune cells into the CNS, reducing neuroinflammation and neuronal damage.

## Neutrophils’ influence on neuronal function

Neutrophils, traditionally recognized for their role in the immune system, are increasingly understood to have significant impacts on neuronal function. These cells, which are the most abundant type of white blood cells, play a critical role in inflammation and immune responses. In the context of schizophrenia, a complex psychiatric disorder, the influence of neutrophils on neuronal function has gained attention due to its potential implications for understanding the disorder’s pathophysiology and developing new therapeutic approaches. While the central nervous system (CNS) was once considered an immune-privileged site, it is now clear that immune cells, including neutrophils, can interact with neural tissues^[29]^. Under normal conditions, the blood–brain barrier (BBB) restricts the entry of peripheral immune cells into the CNS. However, during inflammation or immune activation, neutrophils can traverse the BBB and infiltrate the CNS. This infiltration can have both protective and detrimental effects on neuronal function, depending on the context and extent of the immune response. Neuroinflammation is a critical factor in the pathogenesis of schizophrenia. Neutrophils contribute to this process by releasing proinflammatory cytokines, chemokines, and reactive oxygen species (ROS). These substances can lead to neuronal damage and dysfunction. For instance, cytokines such as interleukin-6 (IL-6) and tumor necrosis factor-alpha (TNF-α) can disrupt synaptic function and plasticity, impairing cognitive processes. ROS, on the other hand, can cause oxidative damage to neurons, affecting their viability and function^[30]^.

Neutrophils generate ROS as part of their antimicrobial activities. In schizophrenia, excessive ROS production by neutrophils can lead to oxidative stress, which is characterized by an imbalance between ROS production and the body’s ability to detoxify these reactive intermediates. Oxidative stress can damage neuronal cell membranes, mitochondria, and DNA, leading to impaired neuronal function and cell death. This damage is associated with cognitive deficits and other symptoms of schizophrenia, highlighting the importance of managing oxidative stress in therapeutic strategies^[26]^. The integrity of the BBB is crucial for maintaining CNS homeostasis. Neutrophils can disrupt the BBB through the release of proteolytic enzymes and ROS. This disruption increases BBB permeability, allowing more immune cells and inflammatory mediators to enter the CNS. The resulting increase in neuroinflammation can exacerbate neuronal damage and dysfunction, contributing to the clinical manifestations of schizophrenia. Studies have shown that patients with schizophrenia often exhibit increased BBB permeability, which may be partly driven by neutrophil activity^[27]^. Neutrophils can influence synaptic plasticity, which is essential for learning and memory. Proinflammatory cytokines released by neutrophils can modulate synaptic strength and plasticity, potentially leading to cognitive impairments. For example, TNF-α has been shown to alter synaptic transmission and plasticity by affecting the expression of synaptic receptors. This modulation can result in the cognitive deficits observed in schizophrenia, such as impaired working memory and executive function.

Genetic studies have identified polymorphisms in genes related to immune function and inflammation in individuals with schizophrenia. These genetic variations can affect neutrophil function and their interaction with neurons. Molecular analyses have revealed altered expression of genes involved in neutrophil activation and function in schizophrenia patients. Understanding these genetic and molecular mechanisms can provide insights into how neutrophils contribute to neuronal dysfunction and identify potential targets for therapeutic intervention^[28]^. The influence of neutrophils on neuronal function in schizophrenia has significant therapeutic implications. Targeting neutrophil activity and the inflammatory pathways they modulate could offer new approaches for treating schizophrenia. Anti-inflammatory agents that specifically inhibit neutrophil function or reduce the release of proinflammatory cytokines may help mitigate neuronal damage and improve cognitive function. Antioxidant therapies aimed at reducing ROS levels and protecting against oxidative damage are also promising strategies for preserving neuronal integrity and function.

## Genetic and environmental factors of neutrophils in schizophrenia

Neutrophils, key players in the innate immune response, have been increasingly implicated in the pathophysiology of schizophrenia. Genetic studies have identified several polymorphisms in immune-related genes that are associated with schizophrenia. These polymorphisms can affect the regulation and function of neutrophils, contributing to the immune dysregulation observed in the disorder. For example, variations in genes such as IL-6, TNF-α, and CRP (C-reactive protein) have been linked to altered inflammatory responses in schizophrenia patients^[29,30]^. Specific genes involved in neutrophil activation, chemotaxis, and ROS production have also been implicated in schizophrenia^[31]^. Polymorphisms in the NADPH oxidase complex, which is crucial for ROS generation in neutrophils, can influence oxidative stress levels in individuals with schizophrenia. Additionally, variations in genes encoding neutrophil granule proteins, such as MPO (myeloperoxidase), can affect the inflammatory potential of neutrophils. Gene-environment interactions play a significant role in the development of schizophrenia. Genetic predispositions to immune dysregulation can be exacerbated by environmental factors, leading to an increased risk of developing the disorder. For instance, individuals with genetic variants associated with heightened inflammatory responses may be more susceptible to the effects of prenatal infections or early-life stressors, which can trigger or amplify immune activation and neuroinflammation.

Prenatal exposure to infections, such as maternal influenza, has been linked to an increased risk of schizophrenia in offspring^[31]^. Infections during critical periods of fetal brain development can activate the maternal immune system, leading to the release of proinflammatory cytokines and affecting fetal brain development. Neutrophils, as key mediators of the immune response, can contribute to this process by amplifying inflammation and oxidative stress. Early-life stress, including trauma and adverse childhood experiences, has been associated with an increased risk of developing schizophrenia^[32]^. Stress can activate the hypothalamic-pituitary-adrenal (HPA) axis and lead to the release of stress hormones, which in turn can modulate immune function. Chronic stress can result in prolonged activation of neutrophils and other immune cells, promoting a state of persistent inflammation and contributing to the pathophysiology of schizophrenia. Exposure to environmental toxins, such as air pollution and heavy metals, has been linked to immune dysregulation and an increased risk of schizophrenia^[33]^. These toxins can induce oxidative stress and inflammation, affecting neutrophil function and promoting neuroinflammation. For example, particulate matter in air pollution can activate neutrophils and other immune cells, leading to the release of proinflammatory cytokines and ROS, which can damage neural tissues and disrupt brain function.

Research suggests that neutrophils in individuals with schizophrenia may exhibit functional abnormalities, such as impaired chemotaxis and reduced phagocytic activity^[34,35]^. These dysfunctions can lead to an inadequate immune response and persistent inflammation. Impaired neutrophil function can also result in an inability to effectively clear pathogens and cellular debris, further contributing to neuroinflammation and neuronal damage. Molecular analyses have revealed altered expression of genes involved in neutrophil activation and function in schizophrenia patients. For example, increased expression of genes related to proinflammatory cytokines and oxidative stress pathways has been observed. These changes in gene expression can enhance the inflammatory potential of neutrophils and promote a state of chronic inflammation, exacerbating the clinical symptoms of schizophrenia. For instance, genetic markers associated with immune dysregulation can help identify individuals at risk for schizophrenia. Additionally, measuring levels of proinflammatory cytokines and ROS in blood samples could provide insights into disease activity and response to treatment. Targeting the specific pathways involved in neutrophil-mediated inflammation and oxidative stress offers potential therapeutic strategies for schizophrenia. Anti-inflammatory agents that inhibit proinflammatory cytokine production or block neutrophil activation may help reduce neuroinflammation and improve symptoms. Antioxidant therapies aimed at reducing ROS levels and protecting against oxidative damage are also promising approaches for preserving neuronal function.

## Therapeutic implications of neutrophils in schizophrenia

Neutrophils play a pivotal role in the innate immune response and contribute to neuroinflammation in schizophrenia^[36]^. Targeting pathways involved in neutrophil activation and inflammatory cytokine release represents a promising therapeutic approach. Anti-inflammatory agents, such as NSAIDs, corticosteroids, and specific cytokine inhibitors (e.g. TNF-α inhibitors), may help dampen excessive immune responses and reduce neuroinflammation associated with schizophrenia. By inhibiting neutrophil activation and their subsequent release of proinflammatory mediators, these agents aim to mitigate neuronal damage and improve overall cognitive function. Neutrophils are major producers of reactive oxygen species (ROS), which can lead to oxidative stress and neuronal damage in schizophrenia. Antioxidant therapies offer potential therapeutic benefits by neutralizing ROS and protecting neurons from oxidative damage. Agents such as N-acetylcysteine (NAC), alpha-lipoic acid, and vitamin C have shown antioxidant properties and may help restore redox balance in individuals with schizophrenia. By reducing oxidative stress, these therapies aim to preserve neuronal integrity and improve cognitive outcomes in affected individuals.

In schizophrenia, disruptions in BBB integrity allow for increased infiltration of neutrophils and other immune cells into the CNS, exacerbating neuroinflammation. Therapeutic strategies aimed at restoring BBB integrity, such as endothelial cell stabilizers and specific matrix metalloproteinase inhibitors, may help prevent immune cell infiltration and reduce neuroinflammation. By preserving BBB function, these therapies aim to mitigate neuronal damage and potentially improve clinical outcomes in individuals with schizophrenia^[26]^. Immunomodulatory therapies that target immune dysregulation and restore immune balance represent another approach to treating schizophrenia. For instance, therapies aimed at enhancing regulatory T cell function or promoting anti-inflammatory cytokine production may help suppress excessive immune responses observed in schizophrenia. Biologic agents that target specific immune pathways involved in disease pathogenesis, such as interleukin-6 (IL-6) inhibitors or monoclonal antibodies against inflammatory cytokines, are under investigation for their potential efficacy in schizophrenia treatment. These therapies aim to modulate immune responses, reduce neuroinflammation, and improve clinical symptoms and functional outcomes in affected individuals^[27]^. Given the heterogeneity of schizophrenia and variability in immune dysregulation among patients, personalized medicine approaches hold promise for optimizing treatment outcomes. Biomarkers associated with neutrophil activation, cytokine profiles, and oxidative stress markers may help identify subgroups of patients who are likely to benefit from specific immunotherapies or antioxidant treatments. Integrating genetic, immunological, and clinical data through precision medicine approaches can enable tailored therapeutic interventions that address individualized disease mechanisms and improve overall treatment efficacy.

## Role of CNS glial cells in neuroinflammation

Neuroinflammation is a key process in various central nervous system (CNS) diseases, including neurodegenerative disorders (e.g. Alzheimer’s disease and Parkinson’s disease), psychiatric conditions (e.g. schizophrenia and depression), and traumatic injuries. In the CNS, glial cells play an essential role in maintaining homeostasis and responding to injury or disease. The primary glial cells involved in neuroinflammation are microglia, astrocytes, and oligodendrocytes. These cells, traditionally seen as supportive cells, are now known to actively participate in neuroinflammatory responses and contribute to both protective and pathological processes in the CNS^[37]^.

### Microglia

Microglia are the resident immune cells of the CNS, constituting the brain’s first line of defense against pathogens and injury. They originate from the yolk sac during early development and are considered the CNS’s equivalent of macrophages. Microglia continuously monitor the CNS environment for signs of infection, injury, or abnormal neuronal activity. Microglia play a dual role in neuroinflammation: they can be either protective or detrimental depending on the context of activation. Microglia maintain homeostasis by clearing cellular debris, dead neurons, and pathogens through phagocytosis^[31,38]^. They also release anti-inflammatory cytokines such as interleukin-10 (IL-10) and transforming growth factor-beta (TGF-β), which help resolve inflammation and promote tissue repair. In response to chronic stress or injury, microglia can adopt a proinflammatory state, releasing cytokines like tumor necrosis factor-alpha (TNF-α), interleukin-1β (IL-1β), and interleukin-6 (IL-6). This activation leads to neuroinflammation and contributes to neuronal damage. Prolonged activation of microglia can result in oxidative stress, synaptic dysfunction, and even neurodegeneration, as seen in conditions such as Alzheimer’s disease. M1 (proinflammatory) microglia secrete proinflammatory mediators and ROS, leading to neurotoxicity. M2 (anti-inflammatory) microglia are involved in tissue repair, neuroprotection, and resolution of inflammation. Chronic or unresolved activation of M1 microglia is thought to play a significant role in diseases such as multiple sclerosis (MS), amyotrophic lateral sclerosis (ALS), and schizophrenia^[39,40]^.

### Astrocytes

Astrocytes are the most abundant glial cells in the CNS, providing structural support, regulating neurotransmitter levels, and maintaining the blood–brain barrier (BBB)^[41]^. They are crucial for nutrient supply to neurons, ion homeostasis, and the regulation of synaptic function. Astrocytes become reactive (a process called astrogliosis) in response to CNS injury, infection, or disease. Reactive astrocytes undergo morphological changes and secrete a variety of signaling molecules that contribute to neuroinflammation. Under pathological conditions, reactive astrocytes can produce cytokines and chemokines such as IL-1β, IL-6, TNF-α, and C-C motif chemokine ligand 2 (CCL2), which amplify the inflammatory response and recruit immune cells to the CNS. Astrocyte-mediated neuroinflammation has been implicated in neurodegenerative diseases, where sustained inflammation leads to BBB disruption, loss of neurons, and synaptic dysfunction. While reactive astrocytes initially protect the CNS by containing damage and promoting repair, chronic astrogliosis can exacerbate neuroinflammation and contribute to scarring (gliosis), limiting neuronal regeneration, and function. In diseases like MS and Alzheimer’s disease, astrocytes become chronically reactive, exacerbating neuroinflammation and promoting neurodegeneration. Astrocytes play a crucial role in maintaining the integrity of the BBB. Inflammatory signaling from reactive astrocytes can compromise the BBB, allowing peripheral immune cells and harmful substances to enter the brain, further perpetuating neuroinflammation^[42]^. A1 (neurotoxic) astrocytes are induced by activated microglia and produce neurotoxic factors that lead to neuronal death. This is commonly observed in neurodegenerative diseases. A2 (neuroprotective) astrocytes promote neuroprotection and repair by releasing antiinflammatory mediators and growth factors such as brainderived neurotrophic factor (BDNF) and fibroblast growth factor (FGF).

### Oligodendrocytes

Oligodendrocytes are responsible for producing myelin, the insulating sheath that covers neuronal axons and facilitates rapid electrical signal transmission. Although primarily involved in myelination, oligodendrocytes also play a role in CNS immune responses. Oligodendrocytes are vulnerable to inflammatory mediators, particularly in diseases like MS, where demyelination is a central feature. In MS, oligodendrocytes are targeted by immune cells, leading to myelin loss and axonal damage. The inflammatory milieu created by microglia and astrocytes accelerates oligodendrocyte death and impairs their ability to remyelinate axons, resulting in progressive neurodegeneration and functional loss. Inflammatory signals can impair the maturation of oligodendrocyte precursor cells (OPCs), which are necessary for remyelination after CNS injury. Dysregulation of OPC function contributes to chronic demyelination, as seen in MS^[43]^.

### Interactions between glial cells in neuroinflammation

Neuroinflammation is often the result of complex interactions between microglia, astrocytes, and oligodendrocytes. These cells communicate via cytokines, chemokines, and other signaling molecules to coordinate immune responses. Activated microglia can induce astrocytes to adopt a reactive phenotype (A1), which in turn releases proinflammatory cytokines that exacerbate microglial activation. This feedforward loop can perpetuate neuroinflammation and neuronal damage. Reactive astrocytes secrete factors that influence oligodendrocyte function. In MS, astrocytes release molecules that inhibit OPC maturation, impairing remyelination and contributing to disease progression^[44]^.

### Therapeutic implications of targeting glial cells

Given the critical role of glial cells in neuroinflammation, therapeutic strategies aimed at modulating glial cell function hold promise for treating neuroinflammatory and neurodegenerative diseases. Potential approaches include drugs that shift microglia from a proinflammatory (M1) to an anti-inflammatory (M2) phenotype, which could help resolve neuroinflammation and protect neurons^[45]^. Controlling the reactivity of astrocytes could reduce chronic inflammation and prevent astrocyte-mediated neurotoxicity. Enhancing the function of oligodendrocytes and OPCs through anti-inflammatory therapies could facilitate remyelination and protect axons in diseases like MS.

## Clinical trials in neuroinflammation: targeting glial cells

Given the crucial role of glial cells in neuroinflammation and their contribution to various CNS diseases, several clinical trials have focused on developing therapies that modulate the activity of these cells. These trials aim to reduce inflammation, promote neuroprotection, and improve outcomes in conditions such as Alzheimer’s disease, multiple sclerosis (MS), schizophrenia, and traumatic brain injury. Below are some key areas where clinical trials are being conducted:

### Microglia-targeted therapies

Microglia play a central role in neuroinflammation by producing proinflammatory cytokines and reactive oxygen species (ROS)^[46]^. Therapeutic approaches aim to modulate their activation state, shifting them from a harmful, proinflammatory (M1) phenotype to a protective, anti-inflammatory (M2) phenotype. Several drugs are being tested to inhibit the overactivation of microglia, thereby reducing neuroinflammation. An antibiotic with anti-inflammatory properties, minocycline has been shown to inhibit microglial activation. It is currently being investigated in clinical trials for neurodegenerative diseases such as Alzheimer’s and Parkinson’s disease. Colony-stimulating factor 1 receptor (CSF1R) is a key receptor involved in the survival and activation of microglia. Inhibiting CSF1R reduces the number of activated microglia. Clinical trials are underway to evaluate the efficacy of CSF1R inhibitors in neurodegenerative diseases like ALS and frontotemporal dementia (FTD). A CSF1R inhibitor, PLX5622, has shown promise in preclinical studies by reducing microglial activation and preserving cognitive function in animal models of neurodegeneration. Ongoing trials are testing its safety and efficacy in human populations.

### Astrocyte-targeted therapies

Astrocytes contribute to neuroinflammation through reactive astrogliosis, which can exacerbate inflammation and neuronal damage. Targeting astrocytes to control their reactivity is a focus of current research. A phosphodiesterase inhibitor with anti-inflammatory properties, ibudilast has been shown to reduce astrocyte activation and decrease neuroinflammation. It is currently in clinical trials for MS and ALS, with promising results in reducing neuroinflammatory markers. Astrocytes regulate the levels of glutamate in the synaptic cleft, and dysregulation can lead to excitotoxicity and neuronal damage. Approved for ALS, riluzole modulates glutamate release and has shown some anti-inflammatory effects. Trials are investigating its potential to reduce astrocyte-mediated neuroinflammation in other conditions, including MS and schizophrenia^[47]^.

### Oligodendrocyte-targeted therapies

In diseases like MS, where oligodendrocytes and myelin are targeted by the immune system, therapies that promote remyelination and protect oligodendrocytes are being investigated. This monoclonal antibody targets LINGO-1, a protein that inhibits oligodendrocyte maturation. By blocking LINGO-1, opicinumab promotes remyelination and is being tested in clinical trials for MS. Another anti-LINGO-1 drug, BIIB033, is under investigation for its ability to enhance oligodendrocyte regeneration and remyelination in neuroinflammatory diseases^[47]^.

### Cytokine and chemokine modulators

Since proinflammatory cytokines such as TNF-α, IL-1β, and IL-6 play significant roles in neuroinflammation, targeting these molecules has been a focus of clinical trials. TNF-α inhibitors, such as etanercept and infliximab, have been successful in treating systemic inflammatory diseases like rheumatoid arthritis^[48]^. Clinical trials are exploring their use in neuroinflammatory conditions such as MS and traumatic brain injury, where excessive TNF-α signaling contributes to neurodegeneration. This drug blocks IL-1β and is used in treating inflammatory conditions such as rheumatoid arthritis. Anakinra is currently being tested in trials for Alzheimer’s disease and traumatic brain injury, where IL-1β is implicated in promoting neuroinflammation and neuronal damage.

### Therapies for schizophrenia and psychiatric disorders

Increasing evidence links neuroinflammation and glial cell dysfunction to psychiatric disorders such as schizophrenia, depression, and bipolar disorder^[49]^. Clinical trials are investigating antiinflammatory treatments as adjunctive therapies in managing these conditions. Drugs like aspirin and celecoxib have been investigated in clinical trials for their potential to reduce neuroinflammation and improve symptoms in schizophrenia. Although the results are mixed, certain subgroups of patients with elevated inflammatory markers have shown benefits. Besides its neuroprotective role in neurodegeneration, minocycline has been studied for its anti-inflammatory effects in schizophrenia. Several trials have reported improvements in negative symptoms and cognitive function in patients treated with minocycline as an adjunct to antipsychotics.

### Gene and cell-based therapies

Recent advances in gene and cell therapy have opened new avenues for modulating neuroinflammation. Mesenchymal stem cells (MSCs) have been shown to have potent immunomodulatory effects and are currently being tested in clinical trials for their ability to reduce neuroinflammation in conditions such as MS, stroke, and spinal cord injury. MSCs can release anti-inflammatory cytokines and promote tissue repair, making them a promising candidate for treating neuroinflammatory diseases^[50]^. CRISPR-Cas9 technology is being explored in preclinical models to modify genes involved in glial cell activation and neuroinflammation. Although not yet in clinical trials, CRISPR-based approaches hold potential for treating genetic causes of glial dysfunction.

### Neuroprotective agents in clinical trials

N-Acetylcysteine (NAC) is an antioxidant that has been shown to reduce oxidative stress and inflammation in the CNS^[51]^. It is being evaluated in clinical trials for schizophrenia, bipolar disorder, and other neuropsychiatric conditions. Cannabinoids, particularly CBD (cannabidiol), have anti-inflammatory properties and are being studied in clinical trials for their potential to reduce neuroinflammation in MS, epilepsy, and neurodegenerative disorders.

## Neurotransmitters and inflammation

Neuroinflammation is increasingly recognized as a critical factor in the pathophysiology of many neurological and psychiatric disorders. This inflammation affects neurotransmitter systems, notably dopamine and glutamate, two key neurotransmitters that regulate mood, cognition, and behavior.

### Inflammation and dopamine

Dopamine is a neurotransmitter crucial for motor control, reward processing, and cognition. Neuroinflammation can disturb dopaminergic signaling through multiple mechanisms, including cytokine release and oxidative stress, contributing to neurological and psychiatric conditions^[52]^. Inflammatory cytokines like TNF-α, IL-1β, and IL-6 can disrupt dopamine synthesis and transmission by altering the function of enzymes involved in dopamine metabolism, such as tyrosine hydroxylase (TH), which is crucial for dopamine synthesis. Specifically: Inflammation can downregulate the expression and activity of TH, reducing the production of dopamine from its precursor, L-tyrosine. Proinflammatory cytokines can affect the expression and sensitivity of dopamine receptors (D1-D5). For instance, increased levels of TNF-α have been shown to reduce the density of D2 receptors in the striatum, affecting motor and reward pathways. Inflammatory markers can also increase the activity of the dopamine transporter (DAT), which leads to faster clearance of dopamine from the synapse, resulting in reduced dopaminergic signaling. Inflammation induces oxidative stress in the brain by increasing the production of reactive oxygen species (ROS) and reactive nitrogen species (RNS). Dopaminergic neurons are particularly vulnerable to oxidative damage because dopamine itself can undergo oxidation, forming dopamine quinones that are toxic to neurons. This oxidative damage is linked to the loss of dopaminergic neurons seen in neurodegenerative diseases like Parkinson’s disease. In Parkinson’s, neuroinflammation in the substantia nigra leads to dopaminergic neuron death, contributing to motor symptoms. Elevated levels of proinflammatory cytokines have been found in the cerebrospinal fluid (CSF) and blood of Parkinson’s patients, further supporting the link between inflammation and dopamine dysfunction^[53]^. In schizophrenia, there is evidence that neuroinflammation contributes to the hyperactivity of dopamine pathways. Elevated levels of IL-6 and C-reactive protein (CRP) have been found in patients, suggesting inflammation may exacerbate the dopaminergic imbalance. Anti-inflammatory treatments have been explored as adjunct therapies to antipsychotics, with the goal of reducing excessive dopamine signaling in specific brain regions, like the mesolimbic pathway.

### Inflammation and glutamate

Glutamate is the primary excitatory neurotransmitter in the brain, involved in synaptic plasticity, learning, and memory. Inflammatory processes can dysregulate glutamate transmission, leading to excitotoxicity—a condition where excessive glutamate activity damages neurons^[54]^. Inflammation increases the release of glutamate from neurons and glial cells, contributing to elevated synaptic glutamate levels. Simultaneously, it impairs the function of glutamate transporters on astrocytes, such as EAAT2, reducing the reuptake of glutamate from the synapse. Inflammatory cytokines can modulate the activity of N-methyl-D-aspartate (NMDA) receptors, leading to either hypofunction or hyperfunction, depending on the context. Chronic inflammation often results in NMDA receptor overactivation, contributing to neuronal excitotoxicity, which is a key feature in Alzheimer’s disease and multiple sclerosis (MS).

Microglia, the brain’s resident immune cells, play a central role in neuroinflammation. When activated by cytokines or injury, microglia release large amounts of glutamate, which can overwhelm neurons and contribute to excitotoxic damage. In conditions such as Alzheimer’s and ALS, microglial activation results in excessive glutamate release, which, in combination with impaired glutamate clearance by astrocytes, drives excitotoxicity and neuronal death^[55]^. In schizophrenia, there is growing evidence linking inflammation to glutamatergic dysregulation. Reduced NMDA receptor function is thought to underlie many of the cognitive and negative symptoms of schizophrenia. Inflammatory mediators like IL-6 have been shown to alter NMDA receptor function, contributing to glutamatergic abnormalities in these patients. Astrocytes are key regulators of glutamate levels in the CNS, maintaining synaptic homeostasis through glutamate uptake. Inflammatory conditions impair astrocytic glutamate clearance, further contributing to excitotoxicity. Inflammatory cytokines, such as TNF-α and IL-1β, can downregulate the expression of excitatory amino acid transporter 2 (EAAT2) in astrocytes, resulting in decreased reuptake of glutamate from the synaptic cleft. This creates a toxic accumulation of glutamate, especially in chronic inflammatory states like MS or neurodegenerative diseases. Inflammatory processes have been linked to altered glutamate function in mood disorders like major depressive disorder (MDD) and bipolar disorder^[56]^. Studies have shown increased levels of inflammatory markers in these conditions, correlating with disrupted glutamate neurotransmission. Ketamine, an NMDA receptor antagonist, has rapid antidepressant effects and is thought to work, in part, by normalizing the balance of glutamate transmission. This has led to investigations of anti-inflammatory agents as potential treatments for depression by modulating glutamatergic signaling^[57,58]^.

## Challenges

Neutrophils interact with various immune cells, including microglia, astrocytes, and lymphocytes, within the central nervous system (CNS)^[37]^. These interactions contribute to the complex neuroinflammatory milieu observed in schizophrenia. Schizophrenia is characterized by heterogeneity in clinical presentation, symptom severity, and treatment response^[39]^. Similarly, immune dysregulation, including neutrophil-mediated inflammation, varies among individuals with schizophrenia. This heterogeneity complicates efforts to identify consistent biomarkers or therapeutic targets related to neutrophil function. Addressing this challenge necessitates large-scale studies that stratify patients based on immune profiles and clinical phenotypes. The interplay between genetic predisposition and environmental factors in schizophrenia adds another layer of complexity to studying neutrophils. Genetic variations in immune-related genes influence neutrophil function and inflammatory responses, potentially contributing to disease susceptibility and progression. Environmental factors, such as prenatal infections and psychosocial stressors, further modulate immune dysregulation and may alter neutrophil behavior in susceptible individuals. Integrating genetic and environmental data in studies of neutrophils requires comprehensive longitudinal approaches.

## Future directions

Technological advancements in genomics, proteomics, and single-cell analyses offer unprecedented opportunities to dissect the molecular mechanisms of neutrophil function in schizophrenia^[40]^. High-throughput sequencing and multiomics approaches can identify novel genetic variants, gene expression profiles, and signaling pathways relevant to neutrophil activation and inflammation in the CNS. Utilizing these cutting-edge techniques will enhance our understanding of neutrophil heterogeneity and their contributions to schizophrenia pathogenesis.

Identifying reliable biomarkers associated with neutrophil activation and neuroinflammation is critical for early diagnosis and prognosis in schizophrenia. Biomarkers such as neutrophilto-lymphocyte ratios (NLR), inflammatory cytokine profiles, and oxidative stress markers show promise but require validation in diverse patient cohorts. Integrating biomarker data with clinical assessments may improve diagnostic accuracy, predict treatment response, and facilitate personalized therapeutic strategies targeting neutrophil-mediated inflammation. Developing targeted therapies that modulate neutrophil function and mitigate neuroinflammation represents a promising avenue for schizophrenia treatment. Novel pharmacological agents, including selective cytokine inhibitors, antioxidants, and BBB-stabilizing compounds, are under investigation for their potential to suppress excessive neutrophil activation and preserve neuronal integrity. Clinical trials evaluating these therapies in schizophrenia patients are essential to validate their efficacy, safety, and long-term benefits. Translating findings from preclinical models to clinical settings remains a critical challenge in neutrophil research in schizophrenia. Collaborative efforts between basic researchers, clinicians, and pharmaceutical developers are essential for translating mechanistic insights into effective therapeutic strategies. Multicenter clinical trials incorporating immune biomarkers and advanced imaging techniques can provide robust evidence for the efficacy of neutrophil-targeted therapies in improving patient outcomes.

## Conclusion

The role of neutrophils in schizophrenia represents a significant area of research with profound implications for understanding the disorder’s pathophysiology and developing targeted therapeutic strategies. Neutrophils, traditionally viewed as frontline defenders against infection, are increasingly recognized for their involvement in chronic inflammation and immune dysregulation within the central nervous system (CNS). Neutrophils contribute to neuroinflammation in schizophrenia through the release of proinflammatory cytokines, chemokines, and reactive oxygen species (ROS), which can lead to oxidative stress and neuronal damage. This inflammatory milieu is exacerbated by disruptions in the blood–brain barrier (BBB), allowing neutrophils to infiltrate the CNS and perpetuate neuroinflammation. Genetic and environmental factors play pivotal roles in modulating neutrophil function and immune responses in schizophrenia. Genetic polymorphisms in immune-related genes and environmental exposures, such as prenatal infections and psychosocial stressors, influence susceptibility to the disorder and the severity of immune dysregulation.

## Data Availability

Not applicable as this a review.

## Research registration unique identifying number (UIN)

Not applicable as this a review.
